# Dominance-discovery and discovery-exploitation trade-offs promote diversity in ant communities

**DOI:** 10.1371/journal.pone.0209596

**Published:** 2018-12-31

**Authors:** Louise van Oudenhove, Xim Cerdá, Carlos Bernstein

**Affiliations:** 1 Université Côte d’Azur, INRA, CNRS, ISA, France; 2 Estación Biológica de Doñana, CSIC, Sevilla, Spain; 3 Université de Lyon, Université Claude Bernard Lyon 1, CNRS, Laboratoire de Biométrie et Biologie Evolutive, Villeurbanne, France; Uniiversity of Padova, ITALY

## Abstract

In ant communities, species coexist by using different foraging strategies. We developed an adaptive dynamics model to gain a better understanding of the factors that promote the emergence and maintenance of strategy diversity. We analysed the consequences of both interspecific competition and resource distribution for the evolutionary dynamics of social foraging in ants. The evolution of social foraging behaviour was represented using a stochastic mutation-selection process involving interactions among colonies. In our theoretical community, ant colonies inhabit an environment where resources are limited, and only one resource type is present. Colony interactions depend on colony-specific foraging strategies (defined as the degree of collective foraging), resource distribution patterns, and the degree of competition asymmetry. At the ecological timescale, we have created a model of foraging processes that reflects trade-offs between resource discovery and resource exploitation and between resource discovery and ant behavioural dominance. At the evolutionary timescale, we have identified the conditions of competition and resource distribution that can lead to the emergence and coexistence of both collective and individual foraging strategies. We suggest that asymmetric competition is an essential component in the emergence of diverse foraging strategies in a sympatric ant community.

## Introduction

Niche partitioning plays a significant role in species coexistence in plant and animal communities [1, 2, 3, 4, 5]. Ecological theory predicts that species coexist when their niche differentiation is such that they utilise sufficiently distinct resources [6]. However, Andersen [7] suggests that there are “not enough niches” to explain the high species richness of many ant communities. He argues that the majority of ant species are generalists with overlapping requirements, and some field studies have confirmed the existence of niche inclusion [8, 9, 10]. Notwithstanding, within ant communities, co-occurring species often differ with regards to food type [11, 12, 13], food size [14, 15], daily activity rhythms [16, 17, 18, 19, 20], species thermal tolerance [21, 22, 23], nesting site choice [8], and microhabitat use [24, 25]. Additionally, ants display a wide spectrum of foraging strategies. Foragers range from being completely independent (solitary foraging) to retrieving food resources exclusively through cooperative recruitment [26, 27], and sympatric ant species may use different foraging strategies [28].

Foraging strategies are the result of the complex set of behavioural and morphological traits that are best suited to gathering food resources in a particular environment [29]. In social foragers, the selective value of a given strategy strongly relies on the degree of interdependence among individual foragers [30], and elaborate communication mechanisms facilitate cooperative foraging [26, 31, 32]. For example, not all ant species are capable of displaying recruitment behaviour. This ability depends on the species’ morphology and ability to engage in chemical communication [31]. Individual or non-cooperative foraging does not require communication: each ant finds and harvests food on its own [33]. Recruitment, in contrast, requires that successful discoverers alert inactive nestmates. One of the most communication-intensive strategies is mass recruitment, which employs pheromone trails [34]. In the ant literature, individual-foraging species are commonly referred to as “primitive”, as compared to “advanced” mass-recruiting species [31]. However, the evolution of recruitment behaviour in ants remains unclear, and cladistic analysis suggests that an increase in recruitment efficiency has been repeatedly selected for within different ant clades [35, 36]. A broader hypothesis that seems more biological sound is that foraging behaviour and its relative efficiency have been shaped by selection stemming from environmental constraints [36, 37]. Ant foraging strategies appear to have evolved in response to both intrinsic factors, such as colony size (which seems to be positively correlated with the degree of foraging-related communication in many ant species [38, 39, 40]); and external factors, such as resource distribution patterns [41], competition [42, 40], predation [43], abiotic conditions (e.g. temperature [37, 44, 45], and vegetation [46]).

In eusocial insects, social foraging occurs in two steps. First, a resource is discovered. Second, it is exploited. Scouts search for food, gather information about what they find [47], and, if possible, recruit foragers [31]. Recruits remain in the nest, ready to help exploit resources as soon as scouts relay their discoveries. The size of this pool of recruits determines the efficiency of the recruitment process [27, 48]. At the colony level, the challenge is strike an optimal balance between investing in the discovery rate (high ratio of scouts) and investing in the exploitation rate (high ratio of recruits). We called this dilemma the “discovery-exploitation” trade-off. Different theoretical studies on the scout-recruit constraint in social insects have predicted the optimum proportion of scouts needed to maximize a colony’s gross [49] or net gains [50, 51, 52]. They have also indicated that the optimum strategy should strongly depend on resource characteristics. When food items are either small or easy to find, individual foraging is the most efficient strategy (i.e., all foragers are scouts), whereas other kinds of resources may promote social or cooperative foraging [49, 53]. However, to date, theoretical work on this topic has focused on colony-level dynamics [54]. In contrast, empirical studies have emphasized the importance of both resource characteristics and competitive pressure for foraging strategies [55, 56]. The evolution of foraging strategies might thus be strongly context dependent (i.e., depend on the strategies adopted by the various colonies in a given community). Yet, none of the current models takes into account frequency-dependent competition among species.

Competitive trade-offs are often thought to promote diversity in communities [57, 58, 59], and competitive mechanisms can be distinguished according to the types of interactions involved. For example, interactions might be indirect and result from species harvesting the same limited resource, whereas interference competition involves direct physical interactions among organisms [60]. Ants exhibit both indirect and interference competition. Species that successfully defend food against direct competitors are said to be behaviourally dominant, whereas species that lose during such encounters are said to be behaviourally subordinate [61, 62, 63]; however, the latter species have the ability to discover resources rapidly. Indeed, resource discovery and behavioural dominance are negatively correlated [61, 64] and seem to be species-specific traits [65]. This so-called dominance-discovery trade-off might help promote ant species coexistence [66, 67, 68]. However, it is important to note that the dominance hierarchy is also affected by environmental factors such as temperature [28, 23] and resource availability [11, 69], which act to reduce competitive exclusion [70, 7]. Both empirical and theoretical evidence show that the interplay between species-specific foraging behaviour and context-dependent asymmetric competition plays a fundamental role in shaping ant community structure [12, 68]. Adler and colleagues [68] demonstrated that the dominance-discovery trade-off could promote the coexistence of species that differed in their foraging behaviour. Using a model of food patch occupancy dynamics, they characterised species according to resource discovery rate and worker body mass. They then imposed conditions of asymmetric interspecific competition by assuming that slower discoverers were totally dominant over faster discoverers. In this theoretical community, many species with different discovery rates could coexist. They also showed that when the intensity of the competitive trade-off was relaxed to better reflect realistic community dynamics, coexistence was still possible.

However, the pioneering work by Adler et al. [68] left several important unanswered questions related to the proximate and ultimate causes of the dominance-discovery trade-off. First, from a proximate standpoint, what are the mechanisms underlying the trade-off between dominance and discovery abilities? Second, from a functional standpoint, how did evolution promote the establishment of strategy diversity? Third, how might the dominance-discovery trade-off have favoured the emergence of very different foraging strategies? In this study, we aimed to answer these questions by constructing a community-level demographic model that takes into account recruitment processes and in which foraging strategies structure competitive trade-offs. Indeed, since behavioral dominance is positively correlated with numerical dominance (i.e., forager density) [62, 63], we assumed that success in interference competition depends on the number of foragers exploiting a food resource, which in turn depends on the recruitment ability displayed by the ant species. As in previous models of colony-level foraging in social insects, colony energy intake was constrained by the discovery-exploitation trade-off [50, 51, 54]. Since interspecific competition is a key structuring force in ant communities [31, 71], we likewise took frequency-dependent competition into account. We then used our model to examine how the interplay between resource characteristics and species interactions could determine the coexistence of foraging strategies at the ecological timescale and shape foraging strategy dynamics at the evolutionary timescale. To this end, we linked demographic dynamics and foraging processes. Our mathematical expressions were parametrised using field data. We then used adaptive dynamics to test to the extent to which the discovery-exploitation and dominance-discovery trade-offs could explain the emergence and maintenance of foraging strategy diversity in ant communities.

## Modeling

Within colonies, some foragers search for food resources on their own (hereafter, scouts) while others wait in the nest to be recruited (hereafter, recruits). A colony might modulate its investment in scouts versus recruits. In our model, this investment defines the colony’s foraging strategy. The more a colony invests in scouts, the more efficiently it will discover food items. Conversely, a greater investment in recruits means a colony will more efficiently harvest resources (depending on food item size), which could translate into superiority in interference competition [68]. Our aim was to identify evolutionarily stable foraging strategies and to determine the conditions under which such strategies could evolve and coexist, forming a strategy coalition [72]).

We propose a simple model describing the evolution of foraging strategies in ants sharing the same environment. We start by using a set of differential equations to model interactions among ant colonies that are related to competition for food resources. Based on these interactions, we can establish the net rate of energy intake and the fitness of a single colony. Table 1 shows the main parameters used in the model. From this demographic model, and using an adaptive dynamics framework [73, 74, 75, 76], we can deduce the evolution of foraging strategies. We develop functions that explicit the different foraging processes afterwards.

**Table 1:** Parameters used in the model.

Symbol	Description
*x*_*i*_	Degree of collective foraging
*q*_*i*_	Number of colonies using a strategy *x*_*i*_
*R*_*i*_	Number of food items controlled by a colony using a given strategy *x*_*i*_
*D*_*i*_	Discovery rate of a colony using a given strategy *x*_*i*_
*E*_*i*_	Exploitation rate of a colony using a given strategy *x*_*i*_
*C*_*i*,*j*_	Probability that a colony *i* will usurp a food item previously controlled by a colony *j*
*b*	Food item loss rate due to degradation or other organisms
*ρ*	Food item density
*σ*_*R*_ = *ρ*	Renewal rate of food items
*e*_*R*_ = *ρ*^−1^	Amount of energy available per food item
*σ*_*q*_	Efficiency of converting energy into new colonies
*γ*	Colony mortality rate
*μ*	$\text{Rescaled}\;\text{parameter}=\frac{\gamma }{\sigma_{q}}$
*ϕ*	Degree of competition asymmetry
*v*_*D*_	Search speed
*v*_*E*_	Speed of ants carrying food
*l*	Loading capacity
*ψ*	Degree of preemptive competition

Mathematical analyses were performed using Wolfram Mathematica 11.0.

### Demographic model

We treat each colony as an individual, and we assume that all individuals are strictly equivalent except with regards to foraging strategy. In particular, colony size is constant and independent of foraging strategy. Each individual is characterised by its specific strategy (*x*_*i*_), which represents its degree of collective foraging (e.g., the proportion of foragers that are recruits). Hereafter, we will refer to the set of colonies sharing the same strategy *x*_*i*_ as species, and we will denote the number of colonies using this strategy using *q*_*i*_.

In order to simplify computations, we made two main assumptions regarding food availability within the environment. First, food items are randomly distributed with mean density *ρ*. The pace at which new items appear (i.e., their renewal rate *σ*_*R*_) is equivalent to mean food density: *σ*_*R*_ = *ρ*. Second, the total amount of food or biomass available in the environment is fixed: the more numerous the food items (i.e., the higher their density), the smaller their size. The amount of energy available per item *e*_*R*_ is inversely proportional to food density: *e*_*R*_ = *ρ*^−1^.

The first step in understanding an ant community is to focus on resource fate [68]. Let *n* be the number of species sharing the environment. We distinguish (*n* + 1) types of food items based on who controls them: *R* is the number of undiscovered food items, and *R*_*i*_, *i* ∈ [1, *n*] is the number of food items that have been discovered and exploited (i.e., “controlled”) by a given colony of species *i*. A colony loses control of an item by fully harvesting it or by being driven away by a colony of a different species. We assume that the loss of a food item to a colony of the same species is irrelevant to the species-level success of a strategy. Below, we explicitly describe the dynamics of these (*n* + 1) types of food items.

Food-item dynamics are described in equation system (1). Eq (1.a) describes the dynamics of undiscovered food items. These items appear at rate *σ*_*R*_ (because *σ*_*R*_ = *ρ* as stated above) and disappear at rate *b* as they are degraded or consumed by organisms other than ants. The disappearance rate of undiscovered food items (*R*) is the sum of the discovery rates *D*_*i*_ of the *q*_*i*_ colonies of each species. Eq (1.b) describes the dynamics of the food items controlled by a single colony of species *i*. The first term of Eq (1.b) deals with the discovery of new items: a colony of species *i* discovers food items at discovery rate *D*_*i*_. It can discover either as-yet-undiscovered items, *R*, or items controlled by all colonies of species *j*, *q*_*j*_*R*_*j*_, according to probability *C*_*i*,*j*_. The second term of (1.b) corresponds to previously controlled food items that have been fully harvested by a colony or usurped by colonies of other species. Controlled items *R*_*i*_ are harvested at exploitation rate *E*_*i*_ and can be usurped by colonies of species *j* according to probability *C*_*j*,*i*_ and the discovery rate of species *j*, *D*_*j*_. Controlled items also disappear because of non-ant competitors at rate *b*.

$$\begin{array}{c} \left\{ \begin{array}{ccccc} \frac{dR}{dt_{R}} & = & \rho -R(\sum_{i\in [1,n]}q_{i}D_{i}+b) & & \left(.a\right)\\ \frac{dR_{i}}{dt_{R}} & = & D_{i}\left(R+\sum_{j\in [1,n]-\{i\}}C_{i,j}q_{j}R_{j}\right)-R_{i}\left(E_{i}+\sum_{j\in [1,n]-\{i\}}C_{j,i}q_{j}D_{j}+b\right) & ,\forall i\in [1,n] & \left(.b\right) \end{array}\right. \end{array}$$ (1)

System (1) has a unique stable solution: R* and $R_{i}^{*},\forall i\in \left[1,n\right]$ (the existence, uniqueness, and stability of this solution are shown in S1 Appendix). Food item dynamics are much faster than ant species dynamics. Food items change daily, whereas new colonies are established yearly [77]. We can thus consider the number of items controlled by each colony to be a function of foraging ability (*D*_*i*_, *E*_*i*_, ∀*i* ∈ [1, *n*]), colony number (*q*_*i*_, ∀*i* ∈ [1, *n*]), and competitive interference between species (*C*_*i*,*j*_, ∀(*i*, *j*) ∈ [1, *n*]^2^).

The net rate of energy intake for a single colony of species *i* is given by the equilibrium number of food items the colony controls, $R_{i}^{*}$, multiplied by the species’ exploitation rate, *E*_*i*_, and the amount of available energy per item, *e*_*R*_ (*e*_*R*_ = *ρ*^−1^, as mentioned above). This net rate of energy intake is a key component of the fitness Eq (2).

The growth rate of a species depends on the amount of energy its colonies obtain (the net rate of energy intake) and the efficiency with which this energy is converted into new colonies (*σ*_*q*_). Let parameter *γ* be colony mortality. The simplest differential equation describing the dynamics of species *i* is

$$\begin{array}{c} \frac{dq_{i}}{dt}=q_{i}\left(\sigma_{q}\rho^{-1}E_{i}R_{i}^{*}-\gamma \right) \end{array}$$ (2)

In a community with a single resident species *r*, after performing the rescaling transformation *T* = *σ*_*q*_ *t*, Eq (2) becomes

$$\begin{array}{c} \dot{q_{r}}=q_{r}\left(\frac{D_{r}E_{r}}{\left(b+q_{r}D_{r}\right)\left(b+E_{r}\right)}-\mu \right) \end{array}$$ (3)

where $\mu =\frac{\gamma }{\sigma_{q}}$.

If $\mu <\frac{D_{r}E_{r}}{b(b+E_{r})}$, the resident species reaches its asymptotically stable equilibrium number of colonies $q_{r}^{*}=\frac{E_{r}\left(D_{r}-b\mu \right)-b^{2}\mu }{\mu D_{r}\left(E_{r}+b\right)}$. For example, when there is no competition with non-ants (*b* = 0), *μ*^−1^ represents the stable equilibrium number of colonies.

### Evolutionary model

An analysis was carried out at the evolutionary timescale in which the dynamics of a resident-mutant community with two species (*r* and *s*) were characterised. Mutations are assumed to be rare enough to allow species dynamics to stabilise before mutants appear. The resident species *r* begins at its equilibrium number of colonies $q_{r}^{*}$, and the mutant species *s* is initially rare. The resident-mutant system is defined by Eq (2) and system (1). It was used to address the following questions: (1) Will the mutant be able to establish itself in the community?; (2) Will the mutant supplant the resident?; (3) Will both species coexist?; and (4) Will a coalition of two coexisting species resist invasion by a new mutant? The answers to these questions depend on the invasion fitness, which represents the per capita growth rate of an initially rare mutant in a community of residents $F_{r}\left(s\right)$:

$$\begin{array}{c} F_{r}\left(s\right)=\mu \left(\frac{D_{s}E_{s}(\mu {\left(b+E_{r}\right)}^{2}-C_{s,r}\left(b^{2}\mu +\left(b\mu -D_{r}\right)E_{r}\right)}{D_{r}E_{r}(\mu \left(b+E_{s}\right)\left(b+E_{r}\right)-C_{r,s}\left(b^{2}\mu +\left(b\mu -D_{r}\right)E_{r}\right)}-1\right) \end{array}$$ (4)

The long-term evolution of the foraging strategy can be entirely predicted by $F_{r}\left(s\right)$. The slope of the invasion fitness at $x_{s}=x_{r}(\frac{\partial F_{r}\left(s\right)}{\partial x_{s}}{|}_{x_{s}=x_{r}})$, called the selection gradient, describes the direction and speed of evolutionary changes [73, 74, 75, 76]. The values at which the selection gradient vanishes (i.e. the location of the evolutionarily singular strategies or SSs) are the equilibrium points of the evolutionary dynamics described by $x^{*}\;\text{SS}\Leftrightarrow \frac{\partial F_{r}\left(s\right)}{\partial x_{s}}{|}_{x_{s}=x_{r}=x^{*}}=0$. SSs are classified according to their evolutionary and convergence stability [75]. When they are evolutionarily stable ($\frac{\partial F_{r}{\left(s\right)}^{2}}{\partial x_{s}^{2}}{|}_{x_{s}=x_{r}=x^{*}}<0$), no nearby mutants can invade the resident population, and thus the strategy constitutes an evolutionarily stable strategy [78]. SSs are said to be convergence stable ($\frac{\partial F_{r}{\left(s\right)}^{2}}{\partial x_{s}^{2}}{{|}_{x_{s}=x_{r}=x^{*}}+\frac{\partial F_{r}{\left(s\right)}^{2}}{\partial x_{s}\partial x_{r}}|}_{x_{s}=x_{r}=x^{*}}<0$) when they act as evolutionary attractors, which occurs when strategies in the vicinity of an SS evolve towards it [79].

Of particular interest are SSs that are convergence stable but not evolutionarily stable. At these branching points, nearby mutants can coexist with the resident population. The community is therefore composed of two resident species, each characterised by a specific strategy. Mutations might occur in both populations, and a mutant might either replace one of the residents (preserving dimorphism), replace both residents (eliminating dimorphism), or become a third resident (generating diversity). The likelihood that a polymorphic community will evolve depends on the invasion fitness of a scarce mutant in the dimorphic resident community (see [80, 81] for recent developments in branching bifurcation). As in the monomorphic case, the first step is to find the pair of stable resident equilibria ($q_{r_{1}},q_{r_{2}}$). Then both the number of food items exploited by a mutant in such an environment $R_{m}^{*}$ and its invasion fitness are determined. Evolutionary scenarios are evaluated by analysing evolutionary and convergence stability [82].

### Foraging processes

To mathematically describe the discovery-exploitation trade-off and the dominance-discovery trade-off, we needed to explicitly define the functions *D*_*i*_, *E*_*i*_, and *C*_*i*,*j*_. We aimed to establish simple functions that reflect the mechanisms involved in foraging processes and thus make clear the contributions of the co-existing degrees of collective foraging *x*_*i*_ and *x*_*j*_ and food item density *ρ*.

In the simplified environment described above, according to the first assumption, food items have a density of *ρ* (first assumption) and are randomly distributed. Therefore, the mean distance between the nest and the first item encountered is $\frac{1}{2\rho^{\frac{1}{2}}}$ [83], which we felt was a good approximation of the mean distance between the nest and food items in general. The second assumption states that food item size is inversely proportional to food item density (*ρ*^−1^). If food items are treated as two-dimensional, then item diameter can be estimated using $\rho^{-\frac{1}{2}}$.

The foraging functions were built using both logical and phenomenological arguments and parametrised using field data. The study site and species are described in detail in [22], and the fitting methods are explained in S2 Appendix. The data come from an individual forager: *Cataglyphis cursor*. The parameter estimates are listed in Table 2.

**Table 2:** Foraging parameters.

Parameter	Description	Estimate
*v*_*D*_	Search speed (*m*.*s*^−1^) ^a^	0.11
*v*_*E*_	Speed of ants carrying food (*m*.*s*^−1^) ^a^	0.02
*l*	Loading capacity ^b^	1
*ψ*	Degree of preemptive competition^c^	4

^(*a*)^ = estimated from field data (see S2 Appendix for details).

^(*b*)^ = arbitrarily fixed.

^(*c*)^ = estimated from [22].

#### Discovery rate (*D*_*i*_)

**Fig 1:**
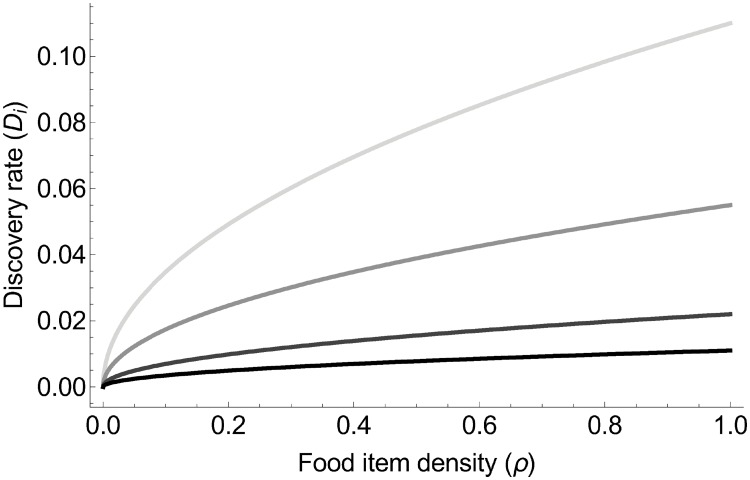
Discovery rate increases with food item density and decreases with the degree of collective foraging *x*. The different colors represent different degrees of collective foraging: light grey for *x* = 0, grey for *x* = 0.5, dark grey *x* = 0.8, and black for *x* = 0.9.

The discovery rate is the number of food items discovered by a given colony per unit time. Between time *t* and *t* + *dt*, a scout explores an area based on its search speed *v*_*D*_, search time *dt*, and food perception abilities [13]. We assume here that the long-distance sensory perception of ants is negligible [84] because most ant species perceive food items upon contact (but see [85]). So, the maximum distance at which an ant perceives a food item corresponds to item diameter: $\rho^{-\frac{1}{2}}$). The simplest approximation of the area explored by a single scout is therefore $v_{D}\rho^{-\frac{1}{2}}dt$. Given that the number of food items per unit area is *ρ*, the number of items encountered by a single scout is $v_{D}\rho^{\frac{1}{2}}dt$. There are (1 − *x*_*i*_) scouts searching for food. We assumed there is no interference among scouts, so the overall discovery rate for a given colony is $D_{i}=v_{D}\rho^{\frac{1}{2}}\left(1-x_{i}\right)$. The rate of discovery thus increases with food item density and decreases with the degree of collective foraging (Fig 1).

#### Exploitation rate (*E*_*i*_)

The exploitation rate represents the fraction of each controlled food item that is consumed per unit time. It is the inverse of the time needed to consume a single food item: *T*_*E*_. It depends on the round-trip travel time between the nest and the food item, *T*_*V*_; the number of trips needed to fully harvest the food item, *N*_*V*_; and the number of ants harvesting the food item, *N*_*A*_: $T_{E}=\frac{T_{V}N_{V}}{N_{A}}$. Travel time is distance divided by speed. The mean distance between a food item and the nest is approximated using $\frac{1}{2\rho^{\frac{1}{2}}}$. Consequently, $T_{V}=\frac{1}{2\rho^{\frac{1}{2}}v_{E}}$, where *v*_*E*_ is the speed of ants carrying food. The number of trips needed to harvest a food item is inversely proportional to ant loading capacity, *l*. Assuming that only the smallest food items can be carried by a single ant in a single load, *l* has a fixed value of 1. Since food item size is proportional to *ρ*^−1^, the food item is fully harvested after $N_{V}=\frac{\rho^{-1}}{l}=\rho^{-1}$ trips.

Regarding the number of foraging ants, we assume that individual foragers remember the position of a food item they have discovered and are able to return to it after delivering a food load to the nest. There is always at least one ant harvesting a given food item. The other ants harvesting the food item have been recruited from the colony’s *x*_*i*_ potential recruits. We consider that the recruits are uniformly distributed across the different food items being harvested. We used the number of food items encountered as a proxy for the number of food items being harvested. Since the number of food items discovered is proportional to $\rho^{\frac{1}{2}}$, the number of recruits available to harvest a given item is proportional to $x_{i}\rho^{\frac{-1}{2}}$. However, not all the potential recruits are immediately engaged in resource harvesting upon resource discovery: the ant trail that allows recruitment requires the pheromone deposits of several ants to be efficient. Therefore, the number of recruits involved depends on the number of trips needed to harvest a given food item: if a large number of trips are needed, the trail has effectively been laid and $x_{i}\rho^{\frac{-1}{2}}$ is a good approximation of the number of effective recruits per food item. If few or no trips are needed, the number of effective recruits also tends towards zero (the food item is fully harvested before recruitment has become efficient). The number of effective recruits per food item can then be modeled using the asymptotic function $x_{i}\rho^{\frac{-1}{2}}\left(1-\frac{1}{N_{V}}\right)$. To summarise, there are $1+x_{i}\rho^{\frac{-1}{2}}\left(1-\rho \right)$ ants harvesting a given food item: the ant that discovered the food item and that kept harvesting it and the ants that were recruited to harvest it.

**Fig 2:**
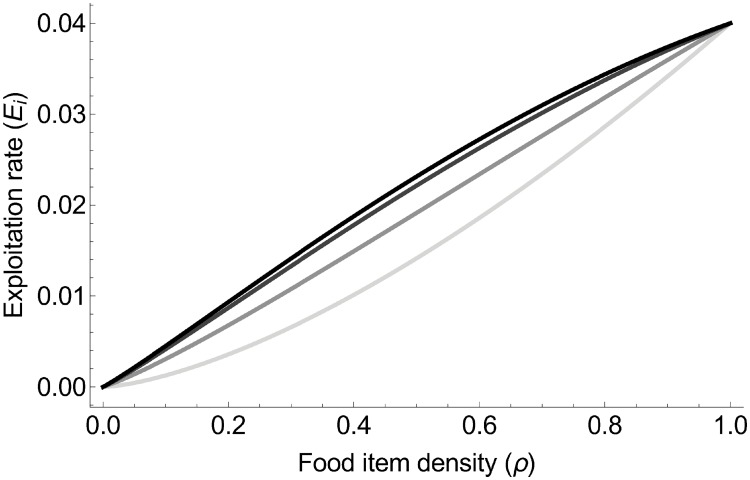
Exploitation rate increases with both food item density and the degree of collective foraging *x*. The different colors represent different degrees of collective foraging: light grey for *x* = 0, grey for *x* = 0.5, dark grey *x* = 0.8, and black for *x* = 0.9.

The colony-level exploitation rate is therefore $E_{i}=\frac{1}{T_{E}}=\frac{N_{A}}{T_{V}N_{V}}=2v_{E}\rho^{\frac{3}{2}}\left(1+x_{i}\rho^{\frac{-1}{2}}\left(1-\rho \right)\right)$. Exploitation rate thus increases with food item density, and increases with the degree of collective foraging (Fig 2).

#### Competition (*C*_*i*,*j*_)

We studied the impacts of two different kinds of competition: preemptive competition and inference competition. In preemptive competition, control over resources occurs on a “first come, first served” basis. Such was the case in our theoretical community where, once a colony has discovered a food item, it cannot be taken over by another colony (*C*_*i*,*j*_ = *C*_*j*,*i*_ = 0).

In interference competition, the probability that a colony will win a direct contest over a food item (and thus usurp it) depends on the size of the food item and the degree of collective foraging of each colony. First, the probability that a colony will usurp a food item from another colony decreases with food item size. Indeed, very small items (*ρ* → 1) can be carried by the ant discovering them and will not be usurped by other colonies. We thus assumed that *C*_*i*,*j*_ is proportional to the linear function (1 − *ρ*), so it tends towards 0 when food size is very small. This probability also depends on the colony’s relative investment in collective foraging. The more a colony invests in recruits, the more workers that will be available to fight over contested food items, the higher the probability that the colony will win. We consider that a colony’s numerical advantage is proportional to its number of effective recruits (defined above), such that the physical competitive strength of a colony with strategy *i* is *x*_*i*_. Based on this relationship, *C*_*i*,*j*_ tends towards 1 (resp. 0) for large food items, if *x*_*i*_ − *x*_*j*_ > 0 (resp. *x*_*i*_ − *x*_*j*_ < 0). The magnitude of the difference in foraging strategies is modulated by the coefficient *ϕ*, which signals the asymmetry of competition. If *ϕ* = 0, competition is symmetric and all colonies have the same probability of winning the item regardless of the degree of collective foraging. The higher the value of *ϕ*, the stronger the asymmetry and the greater the impact of the difference in strategies. In ants, the propensity to start a fight depends on the colony level of aggressiveness. This species-specific trait is positively correlated with investment in collective foraging [86]. The probability that two colonies fight thus depends on both degrees of collective foraging: the more collective their foraging strategies, the higher the probability they actually fight. To account for this fact, we multiplied the numerical advantage (*x*_*i*_ − *x*_*j*_) by the global aggressiveness (*x*_*i*_ + *x*_*j*_). To model colony contests, we used the inverse-logit function $\frac{1}{1+\psi \exp^{-\phi \left(x_{i}-x_{j}\right)\left(x_{i}+x_{j}\right)}}$, where *ψ* represents the degree of preemptive competition. Indeed, when *ψ* → ∞, then *C*_*i*,*i*_ → 0. This parameter was estimated from field observations of *Aphaenogaster senilis* showing that about 20% of contests ended in intraspecific usurpation [22] (Table 2).

**Fig 3:**
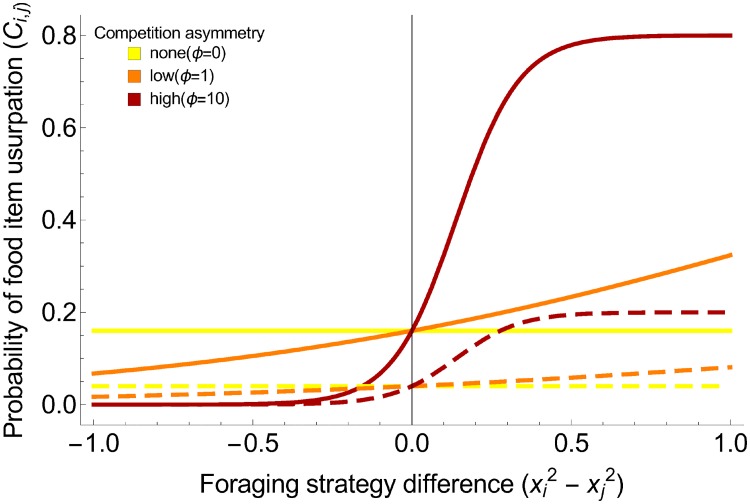
Probability that a colony of species *i* usurps a food item controlled by a colony of species *j* based on the difference in the squared values of their degree of collective foraging ($x_{i}^{2}-x_{j}^{2}$). The yellow lines represent situations in which there is no competitive asymmetry (*ϕ* = 0), the orange lines represent situations of low competitive asymmetry (*ϕ* = 1), and the dark red lines represent situations of high competitive asymmetry (*ϕ* = 10). The dashed lines represent situations in which food items are large and scarce (*ρ* = 0.2), while undashed lines represent situations in which food items are small and abundant (*ρ* = 0.8).

Overall, the probability that a colony *i* will usurp a food item controlled by colony *j* is $C_{i,j}=\frac{1-\rho }{1+\psi \exp^{-\phi (x_{i}^{2}-x_{j}^{2})}}$. The outcome depends on food item size, competition strength, and the degree of collective foraging employed (Fig 3).

### Criteria for strategy emergence and coexistence ($E_{c}$ & $C_{c}$)

The evolution of biodiversity can be analyzed from the branching scenarios [87]. We choose to characterise the diversity of a given community by defining criteria for strategy emergence and coexistence. Competition with non-ant organisms *b* was set to 0.1, and the population parameter *μ* was arbitrarily set to 10^−3^. To ensure the stability of resident populations (see demographic model), values for food item density were constrained (*ρ* ∈ [0.1, 1]).

The emergence criterion $E_{c}$ considers branching events to be opportunities for communities to become more diverse. For a given community, $E_{c}$ was defined as the frequency of parameter values *ρ* (*ρ* ∈ [0.1, 1] discretized to ten decimal places) allowing branching events (see evolutionary model for the definition). For example, a community where a branching event occurred at *ρ* ∈ [0.55, 1] would have a $E_{c}$ of 50%. If $E_{c}=100\%$, then the emergence of diversity is very likely. In contrast, if $E_{c}=0\%$, no alternative foraging strategies are likely to emerge.

The coexistence criterion expresses the likelihood that a pair of strategies will coexist. It is defined as areas of coexistence for a given *ρ* ∈ [0.1, 1] and *ϕ* divided by the sum of total areas for the pair of strategies ((*r*, *s*) ∈ [0, 1]^2^). The areas of coexistence are defined using the pairwise invasibility plots and their mirror images produced along the main diagonal [75]. This serves as a graphic representation of the pairs of strategies that can coexist (i.e., where $F_{r}\left(s\right)>0$ and $F_{s}\left(r\right)>0$) (see evolutionary model, Eq 4 for the definition). To simplify the calculation of the coexistence criterion $C_{c}$, we plotted the pairwise invasibility plot and its mirror image. The plot was binarised; the number of dark pixels was determined and divided by the total number of pixels in a full square (which represents a situation in which all possible paired strategies can coexist) under the same conditions (122,144). $C_{c}$ thus represented the percentage of paired strategies that can coexist. For example, if $C_{c}=0\%$, it means no coexistence was possible, and $C_{c}=100\%$ means that all foraging strategies could coexist.

### Stochastic simulations

To explore how an initially monomorphic community could become more polymorphic, we performed simulations using a stochastic mutation-selection process. We began with a resident community initially composed of a single population at equilibrium that employed a resident strategy *r*; the number of colonies was also at equilibrium ($q_{r}^{*}$; see demographic model for the definition). At each evolutionary time step, a single mutant was introduced into the community (*q*_*m*_ = 1). The mutant’s strategy was a normally distributed random variable whose mean value was the resident strategy and that had a given level of genetic variance ($m∼N(r,var_{G})$). Both resident and mutant populations fluctuated at an ecological timescale (see demographic model for the population dynamics equations). The ecological timescale was considered to be 10^5^ times faster than the evolutionary timescale. If colony number failed to reach non-trivial equilibrium (*q*_*i*_ < 1, *i* ∈ {*r*, *m*}) for a population, that population vanished and its strategy was eliminated from the set of strategies present. If both resident and mutant populations reached a stable non-trivial equilibrium (*q*_*i*_ > 1, ∀*i* ∈ {*r*, *m*}), then they constituted a new resident dimorphic community. In communities composed of several resident populations, one of the resident strategies was randomly chosen to generate a mutant (a single mutant was generated at each time step). As in the the monomorphic community, fluctuation at the ecological timescale determined the equilibrium number of colonies for each population and established whether the new mutant and the residents persisted in the community.

The first set of simulations examined small-scale mutation-selection processes: the parameter value for genetic variance was low (*var*_*G*_ = 0.01). The second set of simulations examined strategies arising from introductions rather than from small-scale mutations; consequently, mutant strategies were not necessarily similar to resident strategies. In this case, genetic variance was not limiting (*var*_*G*_ = 1). As above, competition with non-ant organisms, *b*, had a fixed value of 0.1; the population parameter *μ* was arbitrarily set to 10^−3^.

## Results

### Preemptive competition for food

When competition for food resources in a community is preemptive, it means there is no interference competition (*C*_*i*,*j*_ = *C*_*j*,*i*_ = 0): the first colony to discovers a food item controls it. Two situations can be distinguished.

In the first, there is no competition by non-ants (*b* = 0). Selective forces will thus operate to maximize the probability of discovery. Indeed, for singular strategies, the derivative of the discovery rate vanishes ($D_{x}^{\prime }=0$), and the conditions for both evolutionary and convergence stability correspond to a maximisation of the discovery rate ($D_{x}^{\prime \prime }<0$). When we look at the foraging functions described above, we see that there is no singular strategies *sensu stricto* (∄x∈[0,1] where $D_{x}^{\prime }=0$). However, the whole system converges towards individual foraging ($\hat{x}=0$), which optimises food discovery. In this case, individual foraging is the only evolutionarily and convergence stable strategy (independent of environmental conditions, namely resource density).

**Fig 4:**
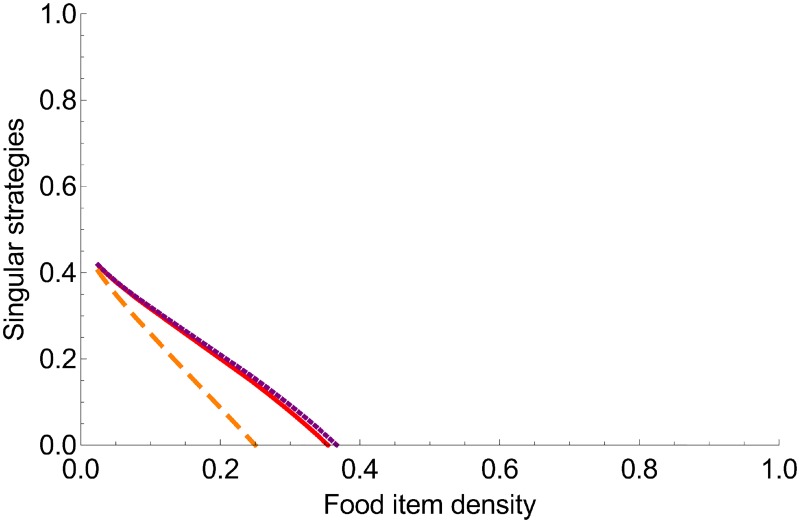
Singular strategies *x* and their stability as a function of the food item density in a community with preemptive competition. The lines represent continuously stable strategies (evolutionarily and convergence stable). The solid red line, dashed orange line and dotted purple line indicate results for *b* = 0.1, *b* = 0.01 and *b* = 0.2, respectively.

When food items are lost to degradation or other organisms (*b* > 0), the selected foraging strategy also depends on foraging processes. For singular strategies *x*, $\frac{D_{x}^{\prime }}{D_{x}}+\frac{bE_{x}^{\prime }}{bE_{x}+E_{x}^{2}}=0$. Using the foraging functions, we show that the value of the singular strategy depends on both food item density (*ρ*) and competition with non-ants (*b*). When food item density is low, the singular strategy is continuously stable (i.e. both evolutionarily and convergence stable). The optimal degree of collective foraging has an intermediate value ($\hat{x}\approx 0.35$ when *b* = 0.1) for the largest food items (*ρ* ≈ 0.1) and then decreases with decreasing food item size; it becomes the individual foraging ($\hat{x}=0$) at the bifurcation value (*ρ*_0_ ≈ 0.35 for *b* = 0.1 or *ρ*_0_ ≈ 0.25 for *b* = 0.01). Increasing the level of non-ant competition (*b*) slightly increases the value of the continuously stable strategy $\hat{x}$ but does not change the general patterns observed (Fig 4). When food item density is high or food item size is small (*ρ* > 0.35 for *b* = 0.1), there is no singular strategy *sensu stricto*, but individual foraging ($\hat{x}=0$) is both convergence and evolutionarily stable.

In both cases, $\frac{\partial F_{r}\left(s\right)}{\partial s}{{|}_{s=r=x}=0\Rightarrow \frac{\partial F_{r}{\left(s\right)}^{2}}{\partial s\partial r}|}_{s=r=x}=0$, meaning that neither emergence nor coexistence of different degrees of collective foraging are possible. A community with preemptive competition for food, a single foraging strategy will become established.

### Strategy-dependent competition

When there is the potential for interference competition, several evolutionary scenarios might result, depending on both food item characteristics (*ρ*) and the degree of competition asymmetry (*ϕ*).

**Fig 5:**
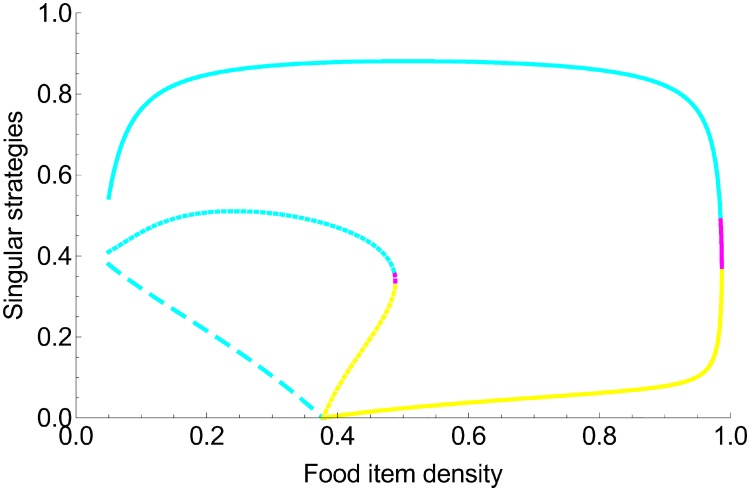
Singular strategies *x* (degree of collective foraging) and their stability as a function of food item density. The cyan lines represent continuously stable strategies (evolutionarily and convergence stable), the yellow lines represent repellors (neither evolutionarily nor convergence stable), and magenta lines represent branching points (evolutionarily unstable and convergence stable). The values and statuses of the singular strategies are plotted for different competition scenarios: the dashed lines represent symmetric competition (*ϕ* = 0); the dotted lines represent weakly asymmetric competition (*ϕ* = 1); and full lines represent strongly asymmetric competition (*ϕ* = 5).

When food items are scarce and large (*ρ* < 0.378 in Fig 5), there is a unique singular strategy that is evolutionarily and convergence stable. Its exact value depends on food item density and the degree of competition asymmetry. When competition is fully symmetric (*ϕ* = 0, Fig 5), it has an intermediate value ($\hat{x}\approx 0.34$) for the largest food items (*ρ* ≈ 0.1), which decreases as food item size decreases. It becomes an individual foraging ($\hat{x}=0$) at the bifurcation value (*ρ*_1_ ≈ 0.378). When competition *ϕ* becomes increasingly asymmetric, the value of the strategy increases (Fig 5). For example, when food items are big and scarce (*ρ* = 0.2), it reaches $\hat{x}\approx 0.5$ when asymmetry is low (*ϕ* = 1) and $\hat{x}\approx 0.85$ when asymmetry is high (*ϕ* = 5).

When food item density is higher than the bifurcation value (*ρ* > 0.378), the evolutionary dynamics strongly depend on the degree of competition asymmetry. When competition is symmetric, there is no singular strategy *sensu stricto*. However, as we found in the case of preemptive competition, long-term dynamics converge toward individual foraging ($\hat{x}=0$), which is evolutionarily stable.

When competition is asymmetric, two other bifurcations appear: *ρ*_2_ ≈ 0.487 and *ρ*_3_ ≈ 0.488 when asymmetry is low (*ϕ* = 1, Fig 5), and *ρ*_2_ ≈ 0.985 and *ρ*_3_ ≈ 0.987 when asymmetry is high (*ϕ* = 5, Fig 5). At food item density *ρ*, such that *ρ*_1_ < *ρ* < *ρ*_2_, the continuously stable singular strategy coexists with an evolutionary repellor (which is neither evolutionarily nor convergence stable). In this case, the evolutionary processes depend on the initial conditions: if the initial strategy has a lower value than the repellor value (${\hat{x}}_{R}\approx 0.1$ for *ρ* = 0.9 and *ϕ* = 5), the system will evolve toward individual foraging; if the initial strategy has a higher value than the repellor, the system will evolve toward collective foraging (${\hat{x}}_{C}\approx 0.8$ for *ρ* = 0.9 and *ϕ* = 5). As food item density increases, the two singular strategies approach each other.

**Fig 6:**
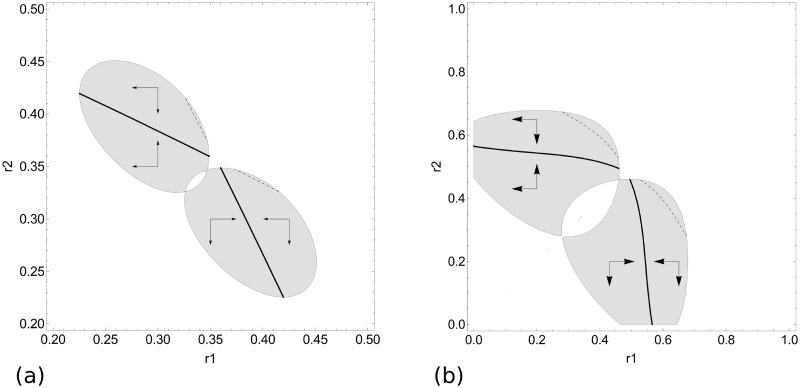
Areas of coexistence and evolutionary isoclines. The grey areas are where there is possible coexistence between strategy *r*_1_ and *r*_2_. The solid lines represent stable isoclines (fitness maxima), whereas the dashed lines represent unstable isoclines (fitness minima). Arrows indicate the direction of evolution. a) a situation where competition asymmetry is low (*ϕ* = 1) and food item density is intermediate (*ρ* = 0.488); b) a situation where competition asymmetry is high (*ϕ* = 5) and food item density is high (*ρ* = 0.986). Here, *b* = 0.1 and *μ* = 0.001.

For food item density *ρ* where *ρ*_2_ < *ρ* < *ρ*_3_, the continuously stable strategy loses its evolutionary stability and becomes a branching point. When the initial strategy value is higher than the repellor value, the evolving strategies progressively increase in value until reaching the branching point. Once there, the evolutionary attractor might be invaded by a nearby mutant. The community thus becomes dimorphic, and the two strategies keep evolving. When competition asymmetry is low (e.g., *ϕ* = 1), evolution moves the strategy outside the range of possible coexistence (Fig 6a). After strategy divergence, both strategies ($x_{r_{1}}\approx 0.15$ and $x_{r_{2}}\approx 0.42$ for *ϕ* = 1) are replaced by a lower-value strategy, and the community becomes monomorphic again. Since this strategy has a much lower value than the repellor, it evolves toward $\hat{x}=0$ (see above). When competition asymmetry is high (e.g., *ϕ* = 5), the two evolving strategies diverge in value until ${\hat{x}}_{r_{1}}=0$ (Fig 6b). Since the pair is evolutionary stable, both strategies coexist (${\hat{x}}_{r_{2}}\approx 0.56$ for *ϕ* = 5). The parameter ranges allowing such an evolutionary scenario are very narrow (*ρ*_3_ − *ρ*_2_ < 0.01, for *ϕ* = 5).

When food item density is higher (*ρ* > *ρ*_3_), evolution results in individual foraging; it is not a singular strategy *sensu stricto* but is both evolutionarily and convergence stable.

#### Emergence and coexistence criteria

**Fig 7:**
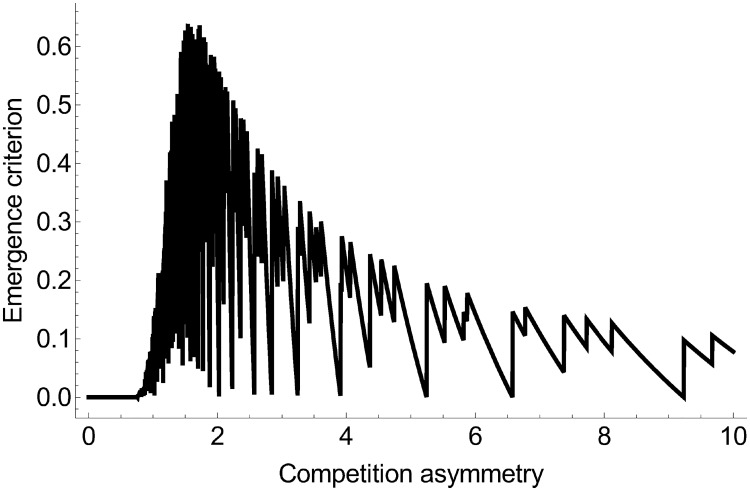
Value of emergence criterion according to the degree of competition asymmetry. Here, *b* = 0.1, *μ* = 0.001, and *ρ* ∈ [0.1, 1].

As mentioned above, under conditions of mutation-selection, only certain circumstances allow the emergence of polymorphism (or the occurrence of branching events) ($E_{c}<1\%$, ∀*ϕ* ∈ [0, 10]). For example, polymorphism was more likely to occur as competition became more asymmetric (Fig 7). However, $E_{c}$ did not increase monotonously with competition asymmetry. Its value was null when competition was symmetric ($E_{c}=0\%$ when *ϕ* < 0.5), it increased until reaching a maximum at an intermediate level of competition asymmetry ($E_{c}\approx 0.5\%$ when *ϕ* ≈ 2), and it then decreased until reaching an equilibrium value when competition was strongly asymmetric ($E_{c}\approx 0.15\%$ when *ϕ* ≈ 8). The emergence criterion is defined as the frequency of *ρ* values that allow branching events. However, a branching event does not necessarily equate to the stable emergence of diversity (e.g. Fig 6a). It must occur in tandem with the potential coexistence of strategies.

**Fig 8:**
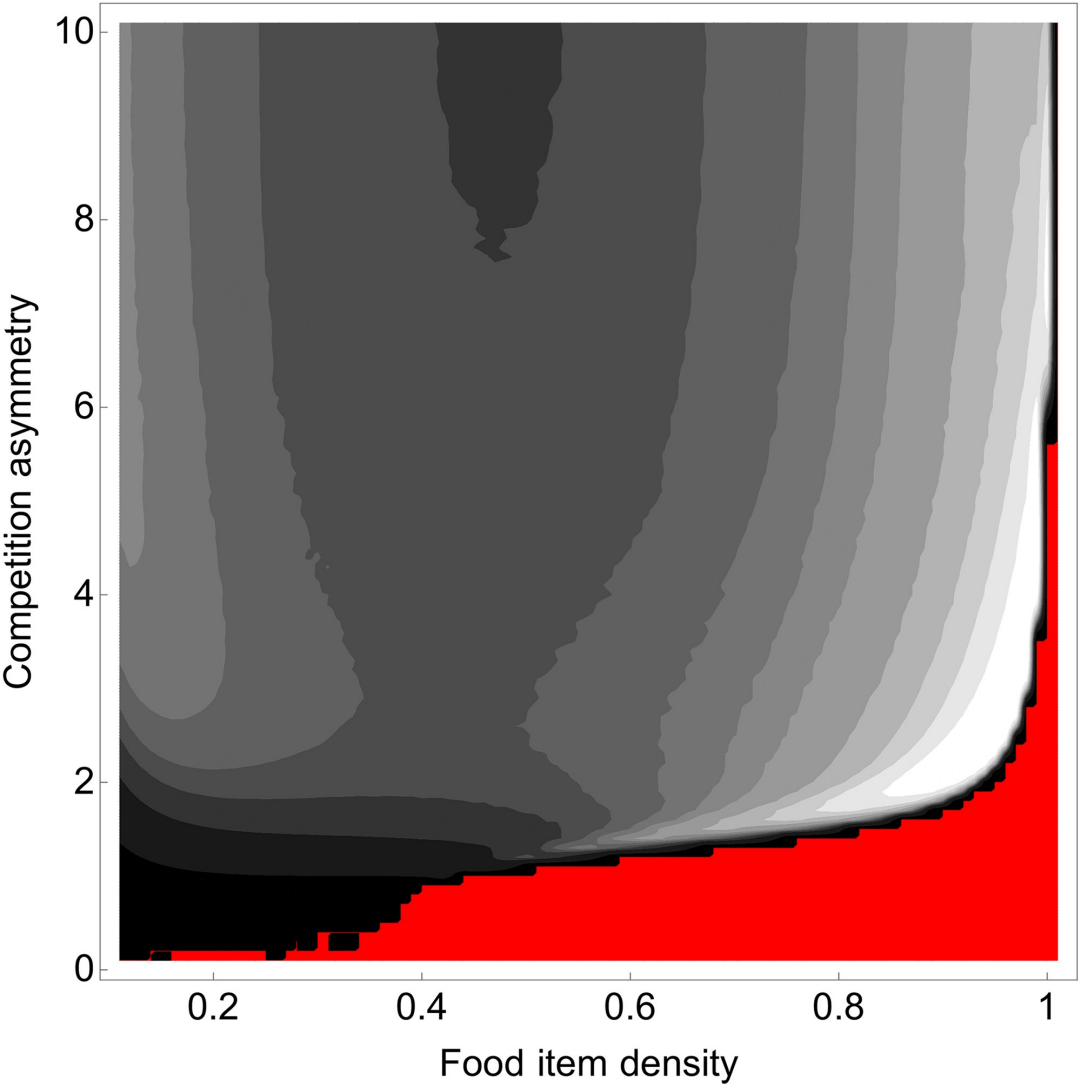
Values for coexistence criterion according to food item density and the degree of competition asymmetry. The red area is where coexistence criterion is zero (no possible coexistence for two different foraging area). The lighter areas indicate where criterion values are higher (i.e. the areas of possible coexistence for two different foraging strategies are larger). Here, *b* = 0.1, *μ* = 0.001.

The value of the coexistence criterion increased as competition became more asymmetric (Fig 8). The potential for coexistence was constrained when competition was weakly asymmetric (e.g., black zone: $C_{c}<1\%$ and red zone $C_{c}=0\%$ when *ϕ* < 1). The range of food densities allowing coexistence increased as the degree of competition asymmetry increased, until coexistence became possible for all types of food items (for *ϕ* > 6). $C_{c}$ is a two-dimensional index that reflects the area of coexistence occupied by two strategies. It took on larger values when competition asymmetry was intermediate and food item density was high (lighter zone in Fig 8, e.g., $C_{c}\approx 30\%$ when *ϕ* = 2 and *ρ* = 0.9).

#### Simulating the emergence of polymorphism

**Fig 9:**
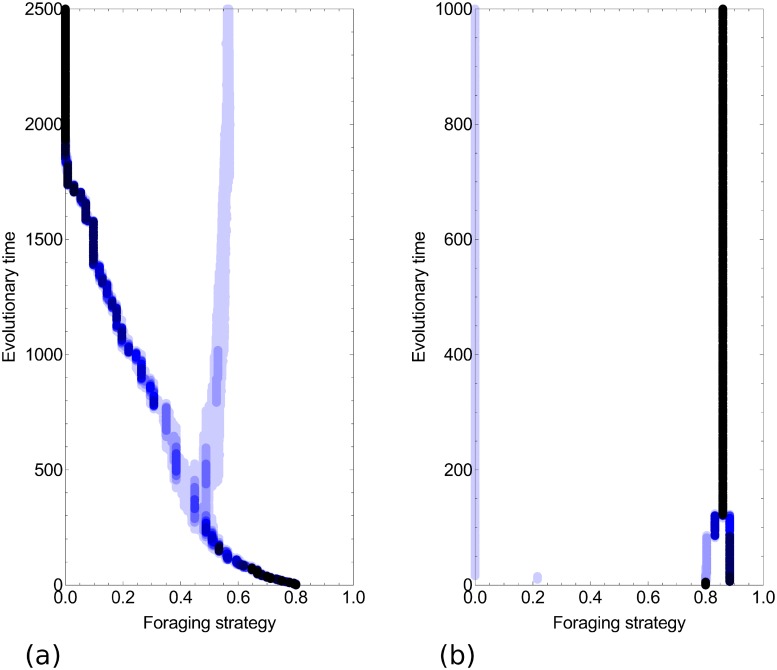
Simulated evolutionary trees for: a) strategies emerging from small-scale mutation-selection processes (*var*_*G*_ = 0.01), where food item density *ρ* = 0.986 and b) strategies emerging from introduction (*var*_*G*_ = 1), where food item density *ρ* = 0.8. Line darkness reflects the relative number of colonies in the population using the corresponding strategy. All the simulations started with a monomorphic community of mass recruiting species (*x*_*r*_ = 0.8). The degree of competition asymmetry *ϕ* = 5. The ecological timescale was assumed to be 10^5^ times faster than the evolutionary timescale. Here, *b* = 0.1 and *μ* = 0.001.

The simulations show how polymorphism can emerge under two scenarios (Fig 9). In the first scenario, strategies evolve via small-scale mutations (i.e., genetic variance is low), and polymorphism emerges after a branching event. The evolutionary tree illustrates that the dimorphic community keeps co-evolving until reaching an equilibrium (*x*_*r*1_ = 0 and *x*_*r*2_ = 0.55 in Fig 9a). These simulations also show that population size is imbalanced: most colonies are individual foragers, while a few are mass recruiters. In the second scenario, the mutant strategy is different from that of the residents because it is introduced from a different community evolving in parallel. The possibilities of coexistence are therefore not strictly tied to branching events. However, similar patterns result because the community rapidly evolves and reaches an equilibrium state where there are individual-foraging colonies and mass-recruiting colonies. In contrast to the mutation-selection scenario, however, most colonies are mass recruiters, while only a few are individual foragers (Fig 9b).

## Discussion

In this study, we explored how colony-level investment in individual versus collective foraging (i.e., scouts vs. recruits) could mechanistically explain the trade-off between food resource discovery and dominance in ants. In a model employing simple functions that was parametrised with field data, we explicitly described two relevant trade-offs: the discovery-exploitation trade-off and the dominance-discovery trade-off. At the evolutionary scale, even though we made the strongly simplistic assumption that the environment contained a single resource type, we showed that the trade-offs allowed strategy diversity. In particular, the degree of competitive asymmetry was a key mediating factor.

Our evolutionary model yielded simple solutions in three different scenarios: when there was no competition (1), when there was symmetric interference competition (2), and when there was asymmetric interference competition (3). (1) In the first (very hypothetical) case of no competition (either from ants or from other organisms), the optimal strategy is to optimize resource discovery via individual foraging: by investing entirely in scouts. (2) In the second case, where there was competition with non-ant organisms and symmetric competition with other ant colonies, two outcomes could be distinguished. (2.1) When resources are scarce and large, the investment in collective foraging should be small. This result might reflect that ants should invest in exploitation to capture resources before they degrade. It could also reflect an investment in recruitment that is just large enough to allow ants to harvest resources that are too large for a single forager to carry [41]. (2.2) When resources are abundant and either medium-sized or small, foraging should be strictly individual. Our conclusions are consistent with those of previous models of the scout-recruit trade-off [50, 49, 51, 52], despite our simpler foraging functions. (3) In the third case, where there was asymmetric competition with other ant colonies, three outcomes could be distinguished. (3.1) When resources are scarce and large, the investment in collective foraging should be great. However, the relative degree of investment depends on the degree of competition asymmetry. The greater the competitive advantage derived from collective foraging, the greater the investment should be. (3.2) When resources are highly abundant and tiny, colonies should invest entirely in individual foraging. (3.3) In intermediate situations (i.e, where food items are medium-sized or somewhat small), individual and collective foraging are evolutionarily stable and can coexist. This last finding fits with those from a previous study examining the association between foraging strategies and spatiotemporal resource attributes, in which both individual and trunk-trail foragers (e.g., leaf-cutting ants) were seen collecting small, common resources [41].

We propose that the investment in collective foraging is the key mechanism underlying the dominance-discovery trade-offs. To make this definition more concrete, we suggest that this investment manifests itself via the trade-off between scouts and recruits, where scouts are workers that search for food by themselves and recruits are workers that harvest food discovered by scouts. However, this vision of foraging can be criticized in two ways. First, ant colonies may display some flexibility in their responses to environmental conditions [43, 41]. They are known to adjust task allocation according to both internal and external factors [88]. In particular, species capable of collective foraging behaviour can efficiently match their efforts to variable environmental characteristics, notably resource distribution [89], resource size [56], resource quality [90], and community composition [91]. Second, this rather black-and-white definition of scouts versus recruits could fail to capture the highly dynamic nature of foraging [92]. In particular, it fails to account for “lost” recruits. Indeed, not all recruits succeed in helping to harvest a given food resource: around 30% of recruits in mass-recruiting species *Tapinoma erraticum* and up to 80% of recruits in group-recruiting species *Tetramorium impurum* fail to reach the target food resource [93]. These “errors” appear to be advantageous since they enhance discovery, especially when resources are highly dispersed and of poor quality [93]. However, here we defined foraging strategy as the relative investment made in collective foraging by a given species, which allows us to avoid these criticisms specifically related to the scout-recruit trade-off. For example, our approach accounts for chemical communication ability. This is a specific trait that would not be modified by plastic behaviours and that would thus preserve the shape of the foraging functions.

There is no direct link between our theoretical model and ant systematics. However, our modeling results underscore that individual foraging would be evolutionarily stable in many different contexts. One such interesting context is when communities are characterised by highly competitive interspecific interactions. It is an outcome that reflects reality since individual-foraging species occur throughout the ant phylogenetic tree [37, 41]. Moreover, in our model, emergence of diversity by means of small-scale mutation-selection processes depends on the ancestral strategy. Unexpectedly, polymorphism could evolve from ancestral collective-foraging strategies but not from ancestral individual-foraging strategies. Even though collective-foraging species are ecologically dominant in ant communities [40], they always coexist with subordinate individual-foraging species [68]. There appears to be enough niche space for both strategies. Furthermore, according to experimental research, the individual-foraging niche is far from being “suboptimal” [94].

Adler et al. [68] demonstrated that the dominance-discovery trade-off could maintain diversity in ant communities. With our evolutionary approach, we have shown that this trade-off coupled with the discovery-exploitation trade-off can also generate diversity. In particular, our results emphasize the role played by asymmetric competition. Diversity was neither generated nor maintained in communities with symmetric competition. Conversely, different strategies were able to evolve in communities with asymmetric competition. Our exploration of the emergence and coexistence criteria revealed that the conditions promoting diversity generally broadened as competition asymmetry increased. The latter reflects the intensity of the dominance-discovery trade-off, which is known to shaped by different selective pressures. First, abiotic conditions such as temperature might influence the outcome of interference competition [14, 23], even though such effects may be context dependent [95]). Second, predators such as parasitoid flies may affect community structure [96, 15, 67]. Experimental research has shown that parasitoids are attracted to the pheromones of their hosts [97]. The trail pheromones laid down by recruits are used as olfactory cues by parasitoids. Both theoretical and empirical evidence indicate that the trade-off between mass recruitment and parasitoid vulnerability reinforces the dominance-discovery trade-off [67]. This so-called “balance of terror” might play a determinant role in shaping competition asymmetry in natural ant communities [98].

The results of this study are consistent with other theoretical predictions underscoring the importance of trade-offs in reducing the potential for competitive exclusion. Negative interactions between life-history traits can occur at different spatial scales and may involve many different factors (see [99] for a review). The trade-offs considered in our model are not specific to ants: their role in community diversity has been discussed in theoretical studies of different organisms. First, the interference-exploitation trade-off seems to allow the coexistence of competitors, regardless of specific resource dynamics [100]. Second, the dominance-discovery trade-off, in which the ability to successfully compete for food stands in opposition to the ability to “colonise” food, is essentially equivalent to the better known competition-colonisation trade-off. The latter is known to promote species coexistence [57] even when competitive intensity is relaxed [59]. Third, the interaction between competitive trade-offs, local dispersal, and species-specific enemies is a mechanism maintaining coexistence in sessile organisms [58]. In short, under conditions of frequency-dependent selection, trade-off shape is crucial in the evolution of life-history traits [101]. It thus seems fundamentally important to understand the allocation constraints that give rise to such trade-offs [102].

Our model involved three strong simplifying assumptions: (i) the environment contains a single resource type whose size is inversely proportional to its abundance; (ii) ant species differ only in their foraging strategies; and (iii) the fitness of ant colonies is proportional to their energy intake. If those assumptions had been relaxed, it probably would have been possible to explain a wider range of foraging strategies. First, a more spatially heterogeneous environment would probably lend itself to a higher degree of diversity, especially for small animals like ants [103]. Models that incorporate environmental complexity, such as variable resource quality, can explore the behavioural flexibility of generalist species, which is known to expand the conditions allowing the coexistence of specialists and generalists within communities [104, 105]. Second, more than 15,916 ant species have been identified [106], and they vary greatly in their biology and ecology. In particular, ant worker body size show substantial intra- (worker polymorphism) and inter-specific variations [26]. The load individual ant workers can transport might thus vary across species, and might also be linked to the competitive ability [40]. More importantly, foraging strategies are associated with colony nest number, activity rhythm, and worker number [40]. Indeed, there is a strong positive correlation between the investment in collective foraging and colony size [38]. As workers are the basis for colony productivity, their numbers are a key part of the population growth rate [26, 107], and they might also acquire adaptations over the course of evolution [39]. Third, there is not necessarily a correlation between foraging activity and reproductive success, especially in ecosystems where abiotic conditions are stressful [108]. In stressful environments, the net rate of foraging intake should be balanced by risk (e.g., desiccation costs in deserts), making it even more probable that different behaviours evolve and coexist [109].

Here, we have identified asymmetric competition as being the cornerstone upon which diversity in social foraging strategies is based in ant communities. Our model is novel because it links small-scale processes, such as the the discovery and exploitation of food resources by a colony to large-scale processes, such as macroscopic evolution at the community level. This approach allowed us to assess the evolutionary dynamics of recruitment strategies and to identify how ant diversity is impacted by such simple factors as food size, food density, and the asymmetry of interspecific competition.

## Supporting information

S1 Appendix Stability of food-item dynamics. (PDF)

S2 Appendix Fitting the model using field data. (PDF)
