# Supplementary material for: Dominance-discovery and discovery-exploitation trade-offs promote diversity in ant communities
Source: PLoS One. 2018 Dec 31;13(12):e0209596. doi: 10.1371/journal.pone.0209596 (PMC6312297; doi:10.1371/journal.pone.0209596)
Supplement: S1 Appendix — (PDF) [file pone.0209596.s001.pdf]

Dominance-discovery and Discovery-exploitation trade-offs promote diversity in ant communities.

Louise van Oudenhove[1,2,3*], Xim Cerdá[2], Carlos Bernstein[3]

[1] Université Côte d'Azur, INRA, CNRS, ISA, France.

[2] Estación Biológica de Doñana, CSIC, Sevilla, Spain

[3] Université de Lyon, Université Claude Bernard Lyon 1, CNRS, Laboratoire de Biométrie et Biologie Evolutive, Villeurbanne, France

* Corresponding author: louise.vanoudenhove@inra.fr

S1 Appendix. Stability of food-item dynamics

The linear system (1) can be written as follows:

$$\dot{\mathbf{x}} = M\mathbf{x} + \mathbf{b}$$

$$\text{where } \mathbf{b} = \begin{pmatrix} \sigma_R \\ 0 \\ \dots \\ 0 \end{pmatrix}, \text{ and M =}$$

$$\begin{pmatrix} -(\sum_{i\in[1,n]} q_i D_i + b) & 0 & 0 & \dots & 0 \\ D_1 & -(E_1 + \sum_{j\in[2,n]} C_{j,1} q_j D_j + b) & C_{1,2} q_2 D_1 & \dots & C_{1,n} q_n D_1 \\ \dots & & & & \\ D_n & C_{n,1} q_1 D_n & C_{n,2} q_2 D_n & \dots & -(E_n + \sum_{j\in[1,n-1]} C_{j,n} q_j D_j + b) \end{pmatrix}$$

Note that M is a Metzler matrix since $\forall i \neq j, m_{i,j} \geq 0$ (Metzler, 1945). System (1) is thus positive and yields a real (and unique) dominant eigenvalue λ_F and a positive associated eigenvector $\mathbf{x}_F$. A necessary and sufficient condition for the linear system (1) to allow a positive and stable solution is that $\lambda_F < 0$ (Farina and Rinaldi, 2011).

$\lambda_0 = -(\sum_{i\in[1,n]} q_i D_i + b)$ is a trivial eigenvalue of M. We assume that there is at least one colony able to discover resources (i.e. $\exists i$ such that $q_i D_i \neq 0$). Since $b \geq 0$, λ_0 is a strictly negative real eigenvalue.

The other eigenvalues of M are the eigenvalues of A_n. We define A_n where $e_i = E_i + b > 0$; $a_{i,j} = C_{i,j} D_i \geq 0$; and $q_i \geq 0$.

$$A_n = \begin{pmatrix} -(e_1 + \sum_{j\in[2,n]} a_{j,1}q_j) & a_{1,2}q_2 & \dots & a_{1,n}q_n \\ \dots & & & \\ a_{n,1}q_1 & a_{n,2}q_2 & \dots & -(e_n + \sum_{j\in[1,n-1]} a_{j,n}q_j) \end{pmatrix}$$

Note that if $\exists I \neq \{\}$ such that $\forall i \in I, q_i = 0$, then $\lambda_i = -(e_i + \sum_{j\neq i} a_{j,i}q_j)$ are strictly negative eigenvalues ($\forall i \in I$) and the remaining eigenvalues are those of the matrix $A_{n-card(I)}$.

Let us now consider A_n where $q_i \neq 0, \forall i \in [1,n]$. A_n's eigenvalues are the λ verifying $det(A_n - \lambda I) = 0$. The following transformations $Row_i \leftarrow q_i Row_i$ and $Column_i \leftarrow \frac{Column_i}{q_i}$ applied to matrix $(A_n - \lambda I)$ preserve the determinant. A_n and B_n thus share the same eigenvalues, where

$$B_n = \begin{pmatrix} -(e_1 + \sum_{j\in[2,n]} a_{j,1}q_j) & a_{1,2}q_1 & \dots & a_{1,n}q_1 \\ \dots & & & \\ a_{n,1}q_n & a_{n,2}q_n & \dots & -(e_n + \sum_{j\in[1,n-1]} a_{j,n}q_j) \end{pmatrix}$$

Note that B_n is a Metzler matrix. Its dominant eigenvalue $\lambda_{F'}$ is real, and $\lambda_{F'} \leq min\{max_i(r_i^+),\ max_i(c_i^+)\}$ where r_i^+ and c_i^+ denote the sums of the elements of the ith row and column (Farina and Rinaldi, 2011). $c_i^+ = -(e_i + \sum_{j\neq i} a_{ji}q_j) + \sum_{j\neq i} a_{ji}q_j < 0, \forall i \in [1,n]$. If $max_i(r_i^+) < max_i(c_i^+)$, then $max_i(r_i^+) < 0$ and $\lambda_{F'} \leq max_i(r_i^+) < 0$. Else, $\lambda_{F'} \leq max_i(c_i^+) < 0$. In all cases, $\lambda_{F'} < 0$.

It follows that $\lambda_F = max\{\lambda_0,\ \lambda_i \forall i \in I,\ \lambda_{F'}\} < 0$, and system (1) has a unique, positive and stable solution.

References

Farina, L., and S. Rinaldi. 2011. Positive linear systems: theory and applications, vol. 50. John Wiley & Sons.

Metzler, L. A. 1945. Stability of multiple markets: the hicks conditions. Econometrica: Journal of the Econometric Society pages 277–292.
