# Supplementary material for: Dominance-discovery and discovery-exploitation trade-offs promote diversity in ant communities
Source: PLoS One. 2018 Dec 31;13(12):e0209596. doi: 10.1371/journal.pone.0209596 (PMC6312297; doi:10.1371/journal.pone.0209596)
Supplement: S2 Appendix — (PDF) [file pone.0209596.s002.pdf]

Dominance-discovery and Discovery-exploitation trade-offs promote diversity in ant communities.

Louise van Oudenhove[1,2,3*], Xim Cerdá[2], Carlos Bernstein[3]

[1] Université Côte d'Azur, INRA, CNRS, ISA, France.

[2] Estación Biológica de Doñana, CSIC, Sevilla, Spain

[3] Université de Lyon, Université Claude Bernard Lyon 1, CNRS, Laboratoire de Biométrie et Biologie Evolutive, Villeurbanne, France

* Corresponding author: louise.vanoudenhove@inra.fr

1 S2 Appendix. Fitting the model using field data

The field data used to estimate the model parameters come from the original database used by Cerdá et al. (1998). These data were collected in Canet de Mar (Barcelona, NE Spain) from 1992 to 1994 by X. Cerdá, S. Cros and J. Retana with the help of J. Bosch, P. Andrés, C. Gené and D. Company. We used data from individual trips made by single *Cataglyphis cursor* workers carrying small or very small prey (e.g., cockroaches or *Drosophila* fly).

The parametrisation of the foraging functions was optimised using R (R Core Team, 2016).

Ant search speed v_D was estimated from ants transporting tiny items such as flies, which were assumed to represent a negligible burden (1 ± 0.2 mg). The mean speed was 0.1068 $m.s^{-1}$ ($sd = 0.05$, $n = 43$); thus 0.11 was considered to be a good estimate for v_D.

The speed of ants loaded down with food items v_E was estimated from ants carrying small items such as cockroaches (78.1 ± 12.1 mg). The mean speed was 0.0164 $m.s^{-1}$ ($sd = 0.01$, $n = 31$); thus 0.02 was considered to be a good estimate for v_E.

References

Cerdá, X., J. Retana, and A. Manzaneda. 1998. The role of competition by dominants and temperature in the foraging of subordinate species in Mediterranean ant communities. Oecologia 117:404–412.

R Core Team. 2016. R: A Language and Environment for Statistical Computing. R Foundation for Statistical Computing, Vienna, Austria.
